# Sensing-Assisted Secure Communications over Correlated Rayleigh Fading Channels

**DOI:** 10.3390/e27030225

**Published:** 2025-02-21

**Authors:** Martin Mittelbach, Rafael F. Schaefer, Matthieu Bloch, Aylin Yener, Onur Günlü

**Affiliations:** 1 Chair of Information Theory and Machine Learning, Technische Universität Dresden, 01062 Dresden, Germany; martin.mittelbach@tu-dresden.de (M.M.); rafael.schaefer@tu-dresden.de (R.F.S.); 2 School of Electrical and Computer Engineering, Georgia Institute of Technology, Atlanta, GA 30332, USA; matthieu.bloch@ece.gatech.edu; 3 Department of Electrical and Computer Engineering, The Ohio State University, Columbus, OH 43210, USA; yener@ece.osu.edu; 4 Information Theory and Security Laboratory (ITSL), Linköping University, 58183 Linköping, Sweden

**Keywords:** secure integrated sensing and communications, physical layer security, secure feedbacked systems, sensing-assisted secure communications, correlated fading for the feedbacked wiretap channel, secure 6G

## Abstract

We consider a secure integrated sensing and communication (ISAC) scenario, where a signal is transmitted through a state-dependent wiretap channel with one legitimate receiver with which the transmitter communicates and one honest-but-curious target that the transmitter wants to sense. The secure ISAC channel is modeled as two state-dependent fast-fading channels with correlated Rayleigh fading coefficients and independent additive Gaussian noise components. Delayed channel outputs are fed back to the transmitter to improve the communication performance and to estimate the channel state sequence. We establish and illustrate an achievable secrecy-distortion region for degraded secure ISAC channels under correlated Rayleigh fading, for which we show that the signal-to-interference-plus-noise is not a sufficient statistic. We also evaluate the inner bound for a large set of parameters to derive practical design insights. The presented results include parameter ranges for which the secrecy capacity of a classical wiretap channel setup is surpassed and for which the channel capacity is approached. Thus, we illustrate for correlated Rayleigh fading cases that our secure ISAC methods can (i) eliminate the need for the legitimate receiver to have a statistical advantage over the eavesdropper and (ii) provide communication security with minimal rate penalty.

## 1. Introduction

Integrating the digital and physical world, envisioned for future communication systems, requires a network to react to changes in real-time through sensing and communication [1]. An example is a millimeter wave (mmWave) integrated sensing and communication (ISAC) system that aims to sense a target by estimating relevant channel parameters to fine-tune the communication scheme [2,3]. There are multiple recent information-theoretic studies of ISAC that extend previous results, such as [4,5]. Focusing on vehicular radar applications for mmWave systems, an information-theoretic model is proposed in [6] for ISAC. In this model, encoded messages are sent over a state-dependent channel with generalized feedback such that the state is only known at the receiver and the feedback is used to improve communication and to estimate the channel state. The rate-distortion region is characterized for independent and identically distributed (i.i.d.) channel states and memoryless ISAC channels with strictly causal channel output feedback. Subsequent works have considered multiple access channels [7], broadcast channels [6], transmitter actions [8], covert communications [9,10], and low-latency scenarios [11,12,13].

As a single modality is used to both communicate with a legitimate receiver and detect a target, the sensing signal may carry sensitive information about the message communicated, which may then be leaked to a target. Since the signal power at the sensed target impacts both the secrecy and sensing performance, there exists a tradeoff between the two [2,14,15,16,17,18]. This tradeoff is characterized in [14] for degraded and reversely-degraded ISAC channels, when the transmitter aims to reliably communicate with the legitimate receiver by using the ISAC channel, estimate the channel state by using the channel output feedback, and keep the message hidden from the target that acts as an eavesdropper. The results in [14] show that it is possible to surpass the secrecy capacity by using the channel output feedback for secure ISAC applications, which strongly contrasts with and significantly improves on classical physical layer security methods.

In this work, we establish an achievable rate region for stochastically degraded secure ISAC channels under bivariate Rayleigh fading by using a Gaussian channel input. Since closed form expressions for this rate region remain elusive, we derive integral expressions from the involved differential entropies, which are amenable to simplified and stable numerical evaluations. Based on the evaluation results, fundamental insights are presented, including, in particular, parameter ranges for which secure-ISAC rates greater than the secrecy capacity can be achieved and for which the channel capacity is approached. Moreover, we provide accurate approximations, which allow easy-to-compute numerical evaluations.

### 1.1. Main Contributions

A summary of the main contributions of this work is as follows:

- We establish an inner bound on the rate region for stochastically degraded secure ISAC channels under bivariate Rayleigh fading by employing a Gaussian input. Our formulation shows how channel-output feedback can be leveraged to significantly improve the secrecy rate, enabling the system to surpass classical secrecy capacity results.
- We derive integral expressions stemming from the involved differential entropies in the achievable rate region. These expressions are amenable to numerically stable and simplified evaluations, facilitating practical performance analysis.
- For some integral expressions in the achievable rate region, we provide closed form solutions in special cases, such as high SNR regime and uncorrelated fading, which significantly simplifies the numerical evaluations.
- We provide fundamental insights into sensing-assisted secure communication systems, including parameter regimes where the achievable secure-ISAC rates can exceed the secrecy capacity and where approaching the channel capacity (i.e., the maximum possible rate without a secrecy constraint) can be possible. We further present accurate approximations that enable straightforward numerical evaluations and guide system design.

### 1.2. Paper Organization

In Section 2, we define the system model and metrics used. In Section 3, we provide the secrecy-distortion regions for correlated fading additive Gaussian noise (AGN) ISAC channels. In Section 4, we evaluate the provided secrecy-distortion regions for Gaussian inputs, which constitutes an achievable rate region. In Section 5, we illustrate the achievable rate regions by numerical calculations and provide the fundamental insights gained from them. In Section 6, we conclude the paper.

### 1.3. Notation

Uppercase letters denote random variables, while their corresponding lowercase letters represent specific realizations. For a continuous random variable *X*, the probability density function (pdf) is denoted as $f_{X}\left(x\right)$ and the cumulative distribution function (cdf) is given by $F_{X}\left(x\right)=\mathrm{Pr}\left[X\leq x\right]$. Calligraphic letters, such as X, indicate sets, with the cardinality of a set given by |X|. $X^{n}$ represents a sequence $X_{1},X_{2},\ldots ,X_{n}$. We represent cov[·,·] as covariance and var[·] as variance, respectively.

(1) $$\begin{array}{c} I_{0}\left(x\right)=\frac{1}{\pi }\int_{0}^{\pi }e^{x\cos(\phi )}d\phi \end{array}$$

denotes the zeroth-order modified Bessel function of the first kind ([19], 10.25.2, 10.32.1).

(2) $$\begin{array}{c} \mathrm{Ei}\left(z\right)=-\int_{-z}^{\infty }\frac{\exp(-t)}{t}\;dt \end{array}$$

represents the exponential integral function ([19], 6.2.5). γ=0.577216… denotes Euler’s constant ([19], 5.2.3). Moreover,

(3) $$\begin{array}{c} \mathrm{erfi}\left(y\right)=-ı\;\mathrm{erf}\left(ıy\right)=-\frac{2ı}{\sqrt{\pi }}\int_{0}^{ıy}\exp\left(-t^{2}\right)\;dt \end{array}$$

denotes the imaginary error function ([19], 7.2.1), and Fqp(a1,…,ap;b1,…,bq;z) represents the generalized hypergeometric function ([19], 16.2.1).

## 2. System Model and Problem Definition

**Figure 1:**
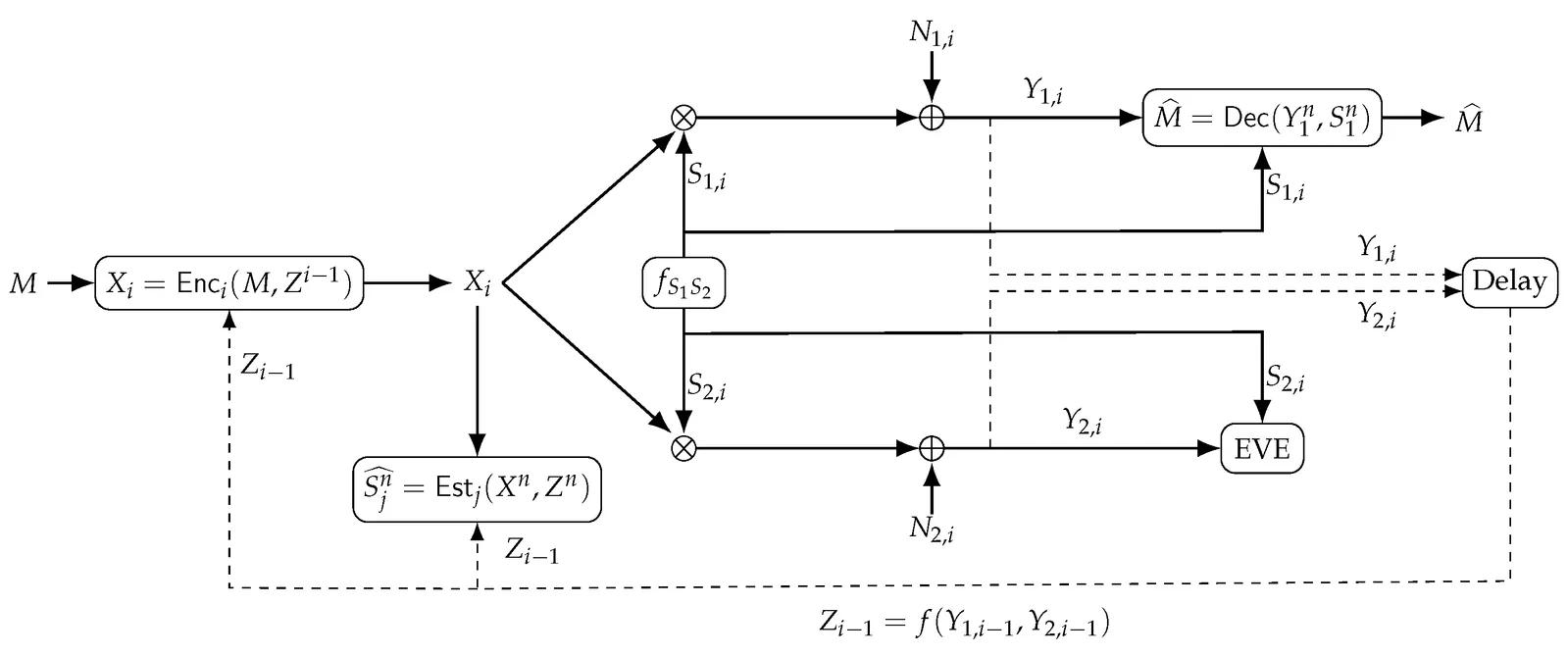
Secure ISAC model for i=[1:n] and j=1,2, for which the message *M* should be kept secret from the eavesdropper. We impose an average transmit power constraint on the channel input symbols $X_{i}$ and assume independent AGN components $N_{1,i}$ and $N_{2,i}$. We principally consider perfect channel output feedback with unit symbol time delay, i.e., $Z_{i-1}=\left(Y_{1,i-1},Y_{2,i-1}\right)$ such that the function f(·,·) is the identity function.

We consider the secure ISAC model depicted in Figure 1, comprising one legitimate receiver, one state estimator, and an eavesdropper (Eve). The transmitter wants to transmit a uniformly distributed message *M* from the finite message set M through a fast fading additive Gaussian noise (AGN) secure ISAC channel, in which i.i.d. fading channel coefficients $\left(S_{1}^{n},S_{2}^{n}\right)$ are causally estimated by the receiver and eavesdropper, respectively. The fading coefficients $\left(S_{1,i},S_{2,i}\right)$ with non-negative real-valued alphabet $S_{1}\times S_{2}$ are correlated according to a known joint pdf $f_{S_{1},S_{2}}$, but their realizations are not known by the transmitter. For discussions about how to extend the results to include complex fading channel coefficients and noise components, see ([20], Section V-A).

Given *M*, the transmitter generates the channel inputs $X^{n}$ by using encoding functions ${\mathrm{Enc}}_{i}\left(\cdot \right)$ such that $X_{i}={\mathrm{Enc}}_{i}\left(M,Z^{i-1}\right)$ for all i=[1:n], where $Z^{i-1}$ is the delayed channel output feedback. We impose an average power constraint on the subsequent transmitted symbols, i.e., we have

$$\begin{array}{c} \frac{1}{n}\sum_{i=1}^{n}E\left[X_{i}^{2}\right]\leq P \end{array}$$

for all messages *M*, where E[·] denotes expectation. The channel output for the legitimate receiver at time *i* is

$$\begin{array}{c} Y_{1,i}=S_{1,i}X_{i}+N_{1,i} \end{array}$$

where $N_{1,i}$ are i.i.d. Gaussian distributed with zero mean, variance $\sigma_{N_{1}}^{2}$, and independent of $\left(S_{1,i},S_{2,i},X_{i}\right)$. The legitimate receiver observes the sequences $\left(Y_{1}^{n},S_{1}^{n}\right)$ and estimates the transmitted message as $\hat{M}=\mathrm{Dec}\left(Y_{1}^{n},S_{1}^{n}\right)$, where Dec(·) is a decoding function. Similarly, the channel output for the eavesdropper at time *i* is

$$\begin{array}{c} Y_{2,i}=S_{2,i}X_{i}+N_{2,i} \end{array}$$

where $N_{2,i}$ are i.i.d. Gaussian distributed with zero mean, variance $\sigma_{N_{2}}^{2}$, and independent of $\left(S_{1,i},S_{2,i},X_{i},N_{1,i}\right)$. The transmitted message *M* should be kept secret from the eavesdropper that observes $\left(Y_{2}^{n},S_{2}^{n}\right)$. Finally, the state estimator observes both the channel output feedback

$$\begin{array}{c} Z_{i-1}=f\left(Y_{1,i-1},Y_{2,i-1}\right) \end{array}$$

and the codeword symbol $X_{i}$ to estimate the fading channel coefficients $\left(S_{1}^{n},S_{2}^{n}\right)$ as $\hat{S_{j}^{n}}={\mathrm{Est}}_{j}\left(X^{n},Z^{n}\right)$ for j=1,2, where ${\mathrm{Est}}_{j}\left(\cdot ,\cdot \right)$ is an estimation function with range $S_{j}^{n}$.

For simplicity, we assume the deterministic processing function f(·,·) is the identity function, so the channel output feedback is perfect, i.e., we have noiseless channel output feedback $Z_{i-1}=\left(Y_{1,i-1},Y_{2,i-1}\right)$. This simplification allows us to obtain fundamental insights into the optimal coding schemes and helps tackle the noisy feedback scenario, which is generally challenging; see, e.g., achievability results for wiretap channels with generalized output feedback in [21]. Note that the achievability proofs for wiretap channels generally require a local randomness source at the encoder, which is true also for the results given below. The randomness can be provided, e.g., by using hardware-intrinsic security primitives [22]. We next define the secrecy-distortion region for the secure correlated fast-fading ISAC problem.

**Definition** **1.** *A secrecy-distortion tuple $\left(R,\;D_{1},\;D_{2}\right)$ is achievable for the secure correlated fast-fading ISAC problem if for any ϵ>0, there exist n≥1, one encoder-decoder pair, and two state estimators ${\mathrm{Est}}_{j}\left(X^{n},Y_{j}^{n}\right)=\hat{S_{j}^{n}}$ such that*

(4) $$\begin{array}{cccc} \; & \mathrm{Pr}[M\neq \hat{M}]\leq \epsilon & \; & \;\;\;\;\;(\mathrm{reliability}) \end{array}$$

(5) $$\begin{array}{cccc} \; & \log|M|\geq n(R-\epsilon ) & \; & \;\;\;\;\;(\mathrm{message}\;\mathrm{rate}) \end{array}$$

(6) $$\begin{array}{cccc} \; & I(M;Y_{2}^{n},S_{2}^{n})\leq n\epsilon & \; & \;\;\;\;\;(\mathrm{secrecy}) \end{array}$$

(7) $$\begin{array}{ccc} E\left[d_{j}\left(S_{j}^{n},\hat{S_{j}^{n}}\right)\right]\;\leq \;D_{j}\;+\;\epsilon \;\;\;\;\;\;\mathrm{for}\;j\;=\;1,2\;\; & \; & \;\;\;\;\;(\mathrm{distortions}) \end{array}$$

*where $d_{j}\left(\cdot ,\cdot \right)$ are averaged per-letter distortion metrics.*

*The secrecy-distortion region $R_{\mathrm{S-ISAC}}$ is the closure of the set of all achievable tuples for the secure correlated fast-fading ISAC problem under perfect channel output feedback.*

Since the transmitted message is independent of the channel state, the secrecy condition in (6) is equivalent to the inequality $I(M;Y_{2}^{n}|S_{2}^{n})\leq n\epsilon$. Furthermore, there are ISAC models, such as in [23], that consider a practical application, in which only a part of the channel parameters are relevant for the transmitter. By not imposing the estimation of the exact channel state at the transmitter via adapting (7), one can extend our results for such practical settings.

## 3. Correlated Fading AGN ISAC Channel Secrecy-Distortion Regions

### 3.1. Secrecy-Distortion Region

We first provide the definition of a degraded ISAC channel; see also [6,14].

**Definition** **2.** *An ISAC channel is* physically degraded *if X and $\left(Y_{2},S_{2}\right)$ are conditionally independent given $\left(Y_{1},S_{1}\right)$. Moreover, an ISAC channel is* stochastically degraded *if the joint probability distribution of $\left(X,Y_{1},S_{1},Y_{2},S_{2}\right)$ can be preserved by using a marginal probability distribution of $\left(X,Y_{1},S_{1}\right)$ such that the corresponding ISAC channel is physically degraded.*

Consider the secrecy-distortion region given in ([14], Theorem 1) for physically degraded secure ISAC channels with discrete-alphabet random variables and state estimators of the form ${\mathrm{Est}}_{j}\left(X^{n},Y_{1}^{n},Y_{2}^{n}\right)=\hat{S_{j}^{n}}$. We evaluate the entropy terms in this rate region to characterize the secrecy-distortion region for the secure ISAC channel considered in this work.

The measures in the secrecy-distortion region in ([14], Theorem 1) remain valid for correlated fading channels with independent AGN components because of the following reasons:

(i) the outer bound applies to arbitrary random variables and does not assume any degradedness;
(ii) there is a discretization procedure to generalize the achievability proof to well-behaved continuous-alphabet random variables, such as the considered fading and noise distributions ([24] Remark 3.8); and
(iii) one can show that changing the estimator form does not change the entropy terms in the rate region, although achieved distortion levels might change since the estimators given in ([14], Theorem 1) should also be adapted.

Moreover, the state estimators considered in Definition 1 make the measures in ([14], Theorem 1) also valid for stochastically degraded channels, which follows because the constraints (4)–(7) in Definition 1 only depend on the marginal probability distributions of $\left(X,Y_{1},S_{1}\right)$ and $\left(X,Y_{2},S_{2}\right)$. This extension is important, as the practical secure ISAC model considered in this work is not physically degraded.

We next consider the secrecy-distortion region for stochastically degraded ISAC channels for our secure ISAC model. Below, expectations with subscripts of random variables indicate that we first calculate the argument of the expectation for fixed realization of the subscript and afterwards calculate the expectation with respect to the distribution of the subscript. Using ([15], Proposition 1), we obtain the following result.

**Corollary** **1.** *The secrecy-distortion region for our secure ISAC model is the union w. r. t. all pdfs $f_{X}$ of the rate-distortion tuples $\left(R,D_{1},D_{2}\right)$ satisfying*

(8a) $$\begin{array}{cc} \; & R\leq \min\{E_{S_{1},S_{2}}\left[h(S_{1}X\;+\;N_{1}|S_{2}X\;+\;N_{2})\right]\;-\;h\left(N_{1}\right) \end{array}$$

(8b) $$\begin{array}{cc} \; & \;\;\;+\;E_{X}\left[h(S_{1}X+N_{1}|S_{2})\right], \end{array}$$

(8c) $$\begin{array}{cc} \; & \;\;\;E_{S_{1}}\left[h(S_{1}X+N_{1})\right]-h\left(N_{1}\right)\}, \end{array}$$

(8d) $$\begin{array}{cc} \; & D_{j}\geq E\left[d_{j}\left(S_{j},{\hat{S}}_{j}\right)\right)]\;\;\;\mathrm{for}\;j=1,2 \end{array}$$

*by using estimators of the form ${\mathrm{Est}}_{j}\left(X^{n},Y_{1}^{n},Y_{2}^{n}\right)=\hat{S_{j}^{n}}$.*

We provide a sufficient but not necessary condition to generate a stochastically degraded secure ISAC channel based on the stochastic ordering of the channel outputs $Y_{1}$ and $Y_{2}$.

**Proposition** **1.** *The considered secure ISAC channel is stochastically degraded if $S_{1}^{2}/\sigma_{N_{1}}^{2}$ is* stochastically larger *than $S_{2}^{2}/\sigma_{N_{2}}^{2}$, i.e., if we have, for all s≥0,*

(9) $${\bar{F}}_{S_{1}^{2}}\;\left(s\;\sigma_{N_{1}}^{2}\right)\geq {\bar{F}}_{S_{2}^{2}}\;\left(s\;\sigma_{N_{2}}^{2}\right).$$

The proof of Proposition 1 follows from ([20], Lemma 3) after appropriate changes to account for the noise variances. For necessity discussions see ([25], Lemmas 1–4).

We next specify the correlated fast-fading distribution, for which we characterize an achievable secrecy-distortion region for stochastically degraded secure ISAC channels.

### 3.2. Bivariate Rayleigh Fading

Suppose the fading random variables $\left(S_{1},S_{2}\right)$ are distributed according to a bivariate Rayleigh fading distribution with pdf $f_{S_{1}S_{2}}\left(s_{1},s_{2}\right)$ equal to

(10) $$\begin{array}{c} \frac{4s_{1}s_{2}}{\sigma_{S_{1}}^{2}\sigma_{S_{2}}^{2}\left(1-\rho^{2}\right)}\exp\left(\;\;-\frac{1}{1-\rho^{2}}\left(\frac{s_{1}^{2}}{\sigma_{S_{1}}^{2}}+\frac{s_{2}^{2}}{\sigma_{S_{2}}^{2}}\right)\right)I_{0}\left(\frac{2}{1-\rho^{2}}\sqrt{\rho^{2}\frac{s_{1}^{2}}{\sigma_{S_{1}}^{2}}\frac{s_{2}^{2}}{\sigma_{S_{2}}^{2}}}\right),\;s_{1},s_{2}\geq 0. \end{array}$$

The parameters $\sigma_{S_{1}}^{2}$ and $\sigma_{S_{2}}^{2}$ in (10) are given as

$$\begin{array}{c} \sigma_{S_{j}}^{2}=E\left[S_{j}^{2}\right],\;j=1,2, \end{array}$$

denoting (with a slight abuse of common notation) the second moments of $S_{1}$ and $S_{2}$. Furthermore, $\rho^{2}$, for $0\leq \rho^{2}<1$, denotes the power correlation coefficient, i.e., we have

$$\begin{array}{c} \mathrm{cor}\left(S_{1}^{2},S_{2}^{2}\right)=\rho^{2}, \end{array}$$

which is the Pearson correlation coefficient between $S_{1}^{2}$ and $S_{2}^{2}$. For later reference, we provide the marginal pdfs $f_{S_{1}}$ and $f_{S_{2}}$ of $S_{1}$ and $S_{2}$ as

(11) $$\begin{array}{cccc} f_{S_{j}}\left(s_{j}\right) & =\frac{2\;s_{j}}{\sigma_{S_{j}}^{2}}\exp\left(-\frac{s_{j}^{2}}{\sigma_{S_{j}}^{2}}\right),\; & & s_{j}\geq 0,\;j=1,2, \end{array}$$

as well as further moments

$$\begin{array}{c} E\left[S_{j}\right]=\sqrt{\frac{\pi }{4}\sigma_{S_{j}}^{2}}, \end{array}$$

$$\begin{array}{c} \mathrm{var}\left[S_{j}\right]=\left(1-\frac{\pi }{4}\right)\sigma_{S_{j}}^{2}, \end{array}$$

$$\begin{array}{c} \mathrm{cov}\left[S_{1},S_{2}\right]\;=\;\sigma_{S_{1}}\sigma_{S_{2}}\left(E\left(\sqrt{\rho^{2}}\right)\;-\;\frac{1}{2}\left(1\;-\;\rho^{2}\right)K\left(\sqrt{\rho^{2}}\right)\;-\;\frac{\pi }{4}\right) \end{array}$$

and

(12) $$\begin{array}{cc} K(z) & =\int_{0}^{\frac{\pi }{2}}{\left(1-z^{2}\sin^{2}\left(t\right)\right)}^{-\frac{1}{2}}\;dt, \end{array}$$

(13) $$\begin{array}{cc} E(z) & =\int_{0}^{\frac{\pi }{2}}{\left(1-z^{2}\sin^{2}\left(t\right)\right)}^{\frac{1}{2}}\;dt \end{array}$$

are the complete elliptic integrals of the first and second kind ([19], 19.2.4, 19.2.5, 19.2.8). Moreover, with the marginals (11) and basic transformations we obtain the complementary cdf of $S_{1}^{2}$ and $S_{2}^{2}$ as

(14) $$\begin{array}{cc} {\bar{F}}_{S_{j}^{2}}\left(s_{j}\right) & =\exp\left(\;-\frac{s_{j}}{\sigma_{S_{j}}^{2}}\right),\;s_{j}\geq 0,\;j=1,2. \end{array}$$

Thus, for the bivariate Rayleigh distribution, the condition (9) on stochastic degradedness is equivalent to

(15) $$\frac{\sigma_{S_{2}}^{2}}{\sigma_{N_{2}}^{2}}\leq \frac{\sigma_{S_{1}}^{2}}{\sigma_{N_{1}}^{2}}.$$

## 4. Achievable Rates for Gaussian Input

Given (8), the main goal is to find the maximum of its right-hand side with respect to the distribution $f_{X}$ of the random variable *X*. However, this is a difficult optimization problem, so we instead provide an achievable rate for a Gaussian input *X*. Subsequently, we evaluate (8a)–(8c) for *X* being a zero-mean Gaussian random variable with positive variance *P*, where *X* is independent of $\left(S_{1},S_{2},N_{1},N_{2}\right)$.

### 4.1. Evaluation of Equation *(8a)*

**Proposition** **2.** *Under the assumptions above, we have*

(16) $$\begin{array}{c} E_{S_{1},S_{2}}\left[h(S_{1}X+N_{1}|S_{2}X+N_{2})\right]-h\left(N_{1}\right)=\frac{1}{2}\int_{0}^{\infty }\log_{2}\left(1+s\right)f_{S}\left(s\right)\;ds \end{array}$$

*where we have*

(17) $$\begin{array}{c} f_{S}\left(s\right)=\sigma_{1}^{2}\sigma_{2}^{2}\exp\left(\frac{\sigma_{2}^{2}}{2P(1-\rho^{2})}\right)\exp\left(-\frac{\sigma_{1}^{2}s+\sqrt{A(s)}}{2P(1-\rho^{2})}\right)\\ \;\;\;\;\times \left(\frac{1}{2P\sqrt{A(s)}}\;+\;\frac{\sigma_{1}^{2}s\;+\;\sigma_{2}^{2}}{2PA(s)}\;+\;\left(1\;-\;\rho^{2}\right)\frac{\sigma_{1}^{2}s+\sigma_{2}^{2}}{A{\left(s\right)}^{\frac{3}{2}}}\right) \end{array}$$

*with*

(18) $$\begin{array}{c} A\left(s\right)={\left(\sigma_{1}^{2}s\right)}^{2}+\left(2-4\rho^{2}\right)\sigma_{1}^{2}\sigma_{2}^{2}s+{\left(\sigma_{2}^{2}\right)}^{2}, \end{array}$$

(19) $$\begin{array}{c} \sigma_{1}^{2}=\frac{\sigma_{N_{1}}^{2}}{\sigma_{S_{1}}^{2}},\;\sigma_{2}^{2}=\frac{\sigma_{N_{2}}^{2}}{\sigma_{S_{2}}^{2}}. \end{array}$$

**Proof** **of Proposition 2.** Fix $S_{1}=s_{1}$ and $S_{2}=s_{2}$ for some $s_{1},s_{2}\geq 0$. Then $\left(s_{1}X+N_{1},s_{2}X+N_{2}\right)$ is jointly Gaussian with zero mean and covariance matrix

(20) $$\begin{array}{c} \left(\begin{array}{cc} s_{1}^{2}P+\sigma_{N_{1}}^{2} & s_{1}s_{2}P\\ s_{1}s_{2}P & s_{2}^{2}P+\sigma_{N_{2}}^{2} \end{array}\right) \end{array}$$

since *X*, $N_{1}$, and $N_{2}$ are independent Gaussian random variables with positive variances *P*, $\sigma_{N_{1}}^{2}$, and $\sigma_{N_{2}}^{2}$. We have

(21) $$\begin{array}{cc} h\left(s_{1}X+N_{1}\;|\;s_{2}X+N_{2}\right) & =h\left(s_{1}X+N_{1},s_{2}X+N_{2}\right)-h\left(s_{2}X+N_{2}\right)\\ & =\frac{1}{2}\log_{2}\left(2\pi e\sigma_{N_{1}}^{2}\right)+\frac{1}{2}\log_{2}\left(1+\frac{s_{1}^{2}/\sigma_{N_{1}}^{2}}{s_{2}^{2}/\sigma_{N_{2}}^{2}+1/P}\right). \end{array}$$

Using (21), we can write

(22) $$\begin{array}{c} E_{S_{1},S_{2}}\left[h(S_{1}X+N_{1}|S_{2}X+N_{2})\right]=h\left(N_{1}\right)+\frac{1}{2}\;E_{S}\left[\log_{2}\left(1+S\right)\right] \end{array}$$

where the random variable *S* is given by

(23) $$\begin{array}{c} S=\frac{T_{1}}{T_{2}+1/P} \end{array}$$

with $\left(T_{1},T_{2}\right)=\left(S_{1}^{2}/\sigma_{N_{1}}^{2},S_{2}^{2}/\sigma_{N_{2}}^{2}\right)$. Since *S* is a ratio of random variables, the pdf $f_{S}$ of *S* has the following integral representation ([26], Equation 6.60):

(24) $$\begin{array}{c} f_{S}\left(s\right)=\int_{1/P}^{\infty }uf_{T_{1},T_{2}}\left(su,u-1/P\right)\;du,\;s\geq 0 \end{array}$$

where $f_{T_{1},T_{2}}$ is the joint pdf of $\left(T_{1},T_{2}\right)$ given by

(25) $$\begin{array}{c} f_{T_{1},T_{2}}\left(t_{1},t_{2}\right)=\;\frac{\sigma_{1}^{2}\sigma_{2}^{2}}{\left(1-\rho^{2}\right)}\exp\left(-\frac{1}{1-\rho^{2}}\left(\sigma_{1}^{2}t_{1}+\sigma_{2}^{2}t_{2}\right)\right)I_{0}\left(\frac{2}{1-\rho^{2}}\sqrt{\rho^{2}\;\sigma_{1}^{2}t_{1}\;\sigma_{2}^{2}t_{2}}\right) \end{array}$$

for $t_{1},t_{2}\geq 0$, where $\sigma_{1}^{2}$ and $\sigma_{2}^{2}$ are as specified in (19).

We substitute $\tilde{u}=\left(u-1/P\right)$ in (24), plug it in (25), and then subsitute $v=\sqrt{\tilde{u}\left(\tilde{u}+1/P\right)\;}$ to obtain

(26) $$\begin{array}{cc} \; & f_{S}\left(s\right)=\frac{\sigma_{1}^{2}\sigma_{2}^{2}}{1-\rho^{2}}\exp\left(\frac{\sigma_{2}^{2}}{2P(1-\rho^{2})}\right)\exp\left(-\frac{\sigma_{1}^{2}s}{2P(1-\rho^{2})}\right)\\ \; & \;\;\times \int_{0}^{\infty }\left[\frac{\theta v}{\sqrt{v^{2}+\theta^{2}}}+v\right]\exp\left(-\alpha \sqrt{v^{2}+\theta^{2}}\right)I_{0}\left(\beta v\right)\;dv \end{array}$$

where we have

(27) $$\begin{array}{c} \alpha =\frac{\sigma_{1}^{2}s+\sigma_{2}^{2}}{1-\rho^{2}},\;\beta =2\frac{\sqrt{\rho^{2}\sigma_{1}^{2}\sigma_{2}^{2}\;s}}{1-\rho^{2}},\;\theta =\frac{1}{2P}. \end{array}$$

Using this representation, we can directly apply ([27], Section 2.15.6.10) and ([27], Section 2.15.6.13). After collecting terms, we finally obtain the form of the density $f_{S}$ given in (17), which completes the proof. □

In the case of uncorrelated fading $\left(\rho^{2}=0\right)$ or for high SNR (P→∞), the representation given in Proposition 2 has the following closed form.

**Corollary** **2.** *(i) For $\rho^{2}=0$, the density *(17)* simplifies to*

(28) $$\begin{array}{c} f_{S}\left(s\right)=\sigma_{1}^{2}\sigma_{2}^{2}\exp\left(\;\;-\frac{\sigma_{1}^{2}s}{P}\right)\left(\frac{1}{P(\sigma_{1}^{2}s+\sigma_{2}^{2})}+\frac{1}{{\left(\sigma_{1}^{2}s+\sigma_{2}^{2}\right)}^{2}}\right) \end{array}$$

*resulting in*

(29) $$\begin{array}{cc} \; & E_{S_{1},S_{2}}\left[h(S_{1}X+N_{1}|S_{2}X+N_{2})\right]-h\left(N_{1}\right)\\ \; & =\left\{\begin{array}{cc} \frac{\sigma_{2}^{2}}{2\ln\left(2\right)\left(\sigma_{1}^{2}-\sigma_{2}^{2}\right)}\left(\exp\left(\frac{\sigma_{1}^{2}}{P}\right)\mathrm{Ei}\left(\;\;-\frac{\sigma_{1}^{2}}{P}\right)-\exp\left(\frac{\sigma_{2}^{2}}{P}\right)\mathrm{Ei}\left(\;\;-\frac{\sigma_{2}^{2}}{P}\right)\right) & \mathrm{if}\;\sigma_{1}^{2}\neq \sigma_{2}^{2}\\ \frac{1}{2\ln(2)}\left(1+\left(\frac{\sigma_{1}^{2}}{P}\right)\exp\left(\frac{\sigma_{1}^{2}}{P}\right)\mathrm{Ei}\left(\;\;-\frac{\sigma_{1}^{2}}{P}\right)\right) & \mathrm{if}\;\sigma_{1}^{2}=\sigma_{2}^{2} \end{array}\right.. \end{array}$$

*(ii) For high SNR (P→∞), the density *(17)* simplifies to*

(30) $$\begin{array}{c} \lim_{P\to \infty }f_{S}\left(s\right)=\sigma_{1}^{2}\sigma_{2}^{2}\left(1-\rho^{2}\right)\frac{\sigma_{1}^{2}s+\sigma_{2}^{2}}{A{\left(s\right)}^{\frac{3}{2}}} \end{array}$$

*with A(s) as in *(18)*, which simplifies for $\sigma_{1}^{2}=\sigma_{2}^{2}$ to*

(31) $$\begin{array}{c} \lim_{P\to \infty }f_{S}\left(s\right)=\frac{\left(1-\rho^{2}\right)\left(s+1\right)}{{\left(s^{2}+\left(2-4\rho^{2}\right)s+1\right)}^{\frac{3}{2}}} \end{array}$$

*resulting in*

(32) $$\begin{array}{cc} E_{S_{1},S_{2}}[h\left(S_{1}X+N_{1}\right|S_{2}X & +N_{2})]-h\left(N_{1}\right)\\ \; & =\frac{1}{4\sqrt{\rho^{2}}}\log_{2}\left(\frac{1+\sqrt{\rho^{2}}}{1-\sqrt{\rho^{2}}}\right)+\frac{1}{4}\log_{2}\left(1-\rho^{2}\right). \end{array}$$

**Proof.**  *Proof of part (i)*: The density (28) follows by (17) for $\rho^{2}=0$. Plugging (28) into (16) and using the substitution t=s+1, we obtain the equivalent integral

(33) $$\begin{array}{c} \int_{1}^{\infty }\frac{\sigma_{1}^{2}\sigma_{2}^{2}}{2}\exp\left(\;\;-\frac{\sigma_{1}^{2}}{P}\left(t-1\right)\right)\log_{2}\left(t\right)\left(\frac{\sigma_{1}^{2}\left(t-1\right)+\sigma_{2}^{2}+P}{P{\left(\sigma_{1}^{2}\left(t-1\right)+\sigma_{2}^{2}\right)}^{2}}\right)\;dt. \end{array}$$

The antiderivative of the integrand in (33) for $\sigma_{1}^{2}\neq \sigma_{2}^{2}$ is given by

(34) $$\begin{array}{c} \begin{array}{c} \frac{\sigma_{2}^{2}}{2\ln\left(2\right)\left(\sigma_{1}^{2}-\sigma_{2}^{2}\right)}\left(\exp\left(\frac{\sigma_{2}^{2}}{P}\right)\mathrm{Ei}\left(\;\;-\frac{\sigma_{1}^{2}\left(t-1\right)+\sigma_{2}^{2}}{P}\right)-\exp\left(\frac{\sigma_{1}^{2}}{P}\right)\mathrm{Ei}\left(\;\;-\frac{\sigma_{1}^{2}}{P}t\right)\right)\\ -\frac{\sigma_{2}^{2}}{2\left(\sigma_{1}^{2}\left(t-1\right)+\sigma_{2}^{2}\right)}\exp\left(\;\;-\frac{\sigma_{1}^{2}}{P}\left(t-1\right)\right)\log_{2}\left(t\right) \end{array} \end{array}$$

which can be obtained by using, e. g., Mathematica. The antiderivative can be directly verified by calculating the derivative of (34) and collecting the terms to re-obtain the integrand of (33). Evaluating (34) for the integration limits of (33), we obtain

(35) $$\begin{array}{cc} t=1:\; & -\frac{\sigma_{2}^{2}}{2\ln\left(2\right)\left(\sigma_{1}^{2}-\sigma_{2}^{2}\right)}\left(\exp\left(\frac{\sigma_{1}^{2}}{P}\right)\mathrm{Ei}\left(\;\;-\frac{\sigma_{1}^{2}}{P}\right)-\exp\left(\frac{\sigma_{2}^{2}}{P}\right)\mathrm{Ei}\left(\;\;-\frac{\sigma_{2}^{2}}{P}\right)\right), \end{array}$$

(36) $$\begin{array}{cc} t\to \infty :\; & 0. \end{array}$$

Substracting (35) from (36) yields the first part of (29).

Similarly, for the antiderivative of the integrand in (33) for $\sigma_{1}^{2}=\sigma_{2}^{2}$, we obtain

(37) $$\begin{array}{c} \begin{array}{c} -\frac{1}{2\ln(2)\;t}\exp\left(\;\;-\frac{\sigma_{1}^{2}}{P}\left(t-1\right)\right)\left(1+\left(\frac{\sigma_{1}^{2}}{P}t\right)\exp\left(\frac{\sigma_{1}^{2}}{P}t\right)\mathrm{Ei}\left(\;\;-\frac{\sigma_{1}^{2}}{P}t\right)\right)-\frac{1}{2t}\log_{2}\left(t\right). \end{array} \end{array}$$

Evaluating (37) for the integration limits of (33), we obtain

(38) $$\begin{array}{cc} t=1:\; & -\frac{1}{2\ln(2)}\left(1+\left(\frac{\sigma_{1}^{2}}{P}\right)\exp\left(\frac{\sigma_{1}^{2}}{P}\right)\mathrm{Ei}\left(\;\;-\frac{\sigma_{1}^{2}}{P}\right)\right), \end{array}$$

(39) $$\begin{array}{cc} t\to \infty :\; & 0. \end{array}$$

Substracting (38) from (39) yields the second part of (29).

*Proof of part (ii)*: The densities (30) and (31) are directly obtained from (17) for P→∞ and $\sigma_{1}^{2}=\sigma_{2}^{2}$. Plugging (31) into (16) and using the substitution t=s+1, we obtain the equivalent integral

(40) $$\begin{array}{c} \int_{1}^{\infty }\frac{\left(1-\rho^{2}\right)\;t\log_{2}\left(t\right)}{2{\left(t^{2}+4\rho^{2}\left(1-t\right)\right)}^{\frac{3}{2}}}\;dt. \end{array}$$

The antiderivative of the integrand in (40) is given by

(41) $$\begin{array}{c} \frac{1}{4}((\frac{1}{\sqrt{\rho^{2}}}+\frac{\left(t-2\right)}{B(t)})\log_{2}\left(t\right)-\frac{1}{\sqrt{\rho^{2}}}\log_{2}\left(\rho^{2}\left(2-t\right)+\sqrt{\rho^{2}}\;B\left(t\right)\right)\\ \;\;\;\;\;\;\;-\log_{2}\left(t-2\rho^{2}+B\left(t\right)\right)) \end{array}$$

where we have $B\left(t\right)=\sqrt{t^{2}+4\rho^{2}\left(1-t\right)}$, and which can be directly verified by calculating the derivative of (41) and collecting the terms to re-obtain the integrand of (40). Evaluating (41) for the integration limits of (40), we obtain

(42) $$\begin{array}{cc} t=1:\; & \frac{1}{4}\left(\log_{2}\left(2(1-\rho^{2})\right)+\frac{1}{\sqrt{\rho^{2}}}\log_{2}\left(\sqrt{\rho^{2}}\left(1+\sqrt{\rho^{2}}\right)\right)\right), \end{array}$$

(43) $$\begin{array}{cc} t\to \infty :\; & \frac{1}{4}\left(1+\log_{2}\left(\sqrt{\rho^{2}}\left(1-\sqrt{\rho^{2}}\right)\right)\right) \end{array}$$

where we use L’Hôpital’s rule to calculate the limit t→∞. Substracting (42) from (43) yields (32). □

### 4.2. Evaluation of Equation *(8b)*

First, we rewrite (8b) as

(44) $$\begin{array}{c} E_{X}\left[h(S_{1}X+N_{1}|S_{2})\right]=E_{X}\left[h(S_{1}X+N_{1},S_{2})\right]-h\left(S_{2}\right). \end{array}$$

Using the marginal pdf of $S_{2}$, we obtain

(45) $$\begin{array}{cc} h(S_{2})= & -\int_{0}^{\infty }f_{S_{2}}\left(s_{2}\right)\log_{2}\left(f_{S_{2}}\left(s_{2}\right)\right)\;ds_{2}\\ = & -2\log_{2}\left(\frac{2}{\sigma_{S_{2}}}\right)\int_{0}^{\infty }u\exp\left(-u^{2}\right)\;du-2\int_{0}^{\infty }u\exp\left(-u^{2}\right)\log_{2}\left(u\right)\;du\\ \; & +\frac{2}{\ln(2)}\int_{0}^{\infty }u^{3}\exp\left(-u^{2}\right)\;du\\ = & \;\frac{1}{\ln(2)}\left(1+\frac{\gamma }{2}\right)+\frac{1}{2}\log_{2}\left(\frac{\sigma_{S_{2}}^{2}}{4}\right) \end{array}$$

using the substitution $u=s_{2}/\sigma_{S_{1}}$ and the integral relations

(46) $$\begin{array}{c} \int_{0}^{\infty }u\exp\left(-u^{2}\right)\;du=\int_{0}^{\infty }u^{3}\exp\left(-u^{2}\right)\;du=\frac{1}{2} \end{array}$$

and

(47) $$\begin{array}{c} \int_{0}^{\infty }u\exp\left(-u^{2}\right)\ln\left(u\right)\;du=-\frac{\gamma }{4}. \end{array}$$

The evaluation of $E_{X}\left[h(S_{1}X+N_{1},S_{2})\right]$ requires the following calculations. Let $Y_{1}\left(x\right)=xS_{1}+N_{1}$. Then, the joint pdf of $\left(Y_{1}\left(x\right),S_{2}\right)$ is given for x>0 by the convolution integral

(48) $$\begin{array}{c} f_{Y_{1}\left(x\right),S_{2}}\left(y_{1},s_{2}\right)\;=\;\int_{0}^{\infty }\frac{1}{x}\;f_{S_{1},S_{2}}\left(\frac{t}{x},s_{2}\right)f_{N_{1}}\left(y_{1}\;-\;t\right)\;dt \end{array}$$

for $-\infty <y_{1}<\infty$ and $s_{2}\geq 0$. Furthermore, we have

(49) $$\begin{array}{c} h\left(xS_{1}+N_{1},S_{2}\right)=-\int_{y_{1}=-\infty }^{\infty }\int_{s_{2}=0}^{\infty }f_{Y_{1}\left(x\right),S_{2}}\left(y_{1},s_{2}\right)\log_{2}\left(f_{Y_{1}\left(x\right),S_{2}}\left(y_{1},s_{2}\right)\right)\;dy_{1}ds_{2}. \end{array}$$

Due to symmetry, we obtain

(50) $$\begin{array}{c} E_{X}\left[h(S_{1}X+N_{1},S_{2})\right]=2\int_{0}^{\infty }h\left(xS_{1}+N_{1},S_{2}\right)f_{X}\left(x\right)\;dx. \end{array}$$

As we can evaluate the convolution integral in (48) numerically, we rely on numerical calculations also for (49) and (50).

An upper bound of $E_{X}\left[h(S_{1}X+N_{1}|S_{2})\right]$, for which the numerical evaluation is much easier, is the following.

**Proposition** **3.** *Under the assumptions above, we have*

(51) EXh(S1X+N1|S2)≤12log22(πe)2σS12c˜P+π2ln(2)erfiσ˜122c˜P+1−1ln(2)σ˜122c˜PF221,1;32,2;σ˜122c˜P+1+γ

*with the parameters*

(52) $$\begin{array}{c} {\tilde{\sigma }}_{1}^{2}=\left(1-\frac{\pi }{4}\right)\frac{\sigma_{N_{1}}^{2}}{\sigma_{S_{1}}^{2}}, \end{array}$$

(53) $$\begin{array}{c} \;\tilde{c}={\left(1-\frac{\pi }{4}\right)}^{2}\left(1-\mathrm{cor}{\left[S_{1},S_{2}\right]}^{2}\right) \end{array}$$

*where we have*

(54) $$\begin{array}{c} \mathrm{cor}\left[S_{1},S_{2}\right]\;=\;\frac{\mathrm{cov}[S_{1},S_{2}]}{\sqrt{\mathrm{var}\left[S_{1}\right]\mathrm{var}\left[S_{2}\right]}}={\left(1-\frac{\pi }{4}\right)}^{-1}\left(E\left(\sqrt{\rho^{2}}\right)-\frac{1}{2}\left(1-\rho^{2}\right)K\left(\sqrt{\rho^{2}}\right)-\frac{\pi }{4}\right) \end{array}$$

*with K(·) and E(·) the elliptic integrals given in *(12)* and *(13)*, respectively.*

**Proof** **of Proposition 3.** Fix X=x for some x∈R. Then, the differential entropy $h\left(xS_{1}+N_{1},S_{2}\right)$ is bounded by

(55) $$\begin{array}{c} h\left(xS_{1}+N_{1},S_{2}\right)\leq \frac{1}{2}\log_{2}({\left(2\pi e\right)}^{2}\left(\mathrm{var}\left[xS_{1}+N_{1}\right]\mathrm{var}\left[S_{2}\right]-\mathrm{cov}{\left[xS_{1}+N_{1},S_{2}\right]}^{2}\right)) \end{array}$$

as a result of the differential entropy maximizing property of the Gaussian distribution with a given covariance matrix. Since $S_{1},S_{2}$, and $N_{1}$ are independent, we have

(56) $$\begin{array}{cc} \; & \mathrm{var}\left[xS_{1}+N_{1}\right]=x^{2}\mathrm{var}\left[S_{1}\right]+\mathrm{var}\left[N_{1}\right], \end{array}$$

(57) $$\begin{array}{cc} \; & \mathrm{cov}\left[xS_{1}+N_{1},S_{2}\right]=x\;\mathrm{cov}\left[S_{1},S_{2}\right]. \end{array}$$

Inserting the definitions of $\mathrm{var}[S_{1}]$ and $\mathrm{cov}\left[S_{1},S_{2}\right]$, we obtain

(58) $$\begin{array}{c} h\left(xS_{1}+N_{1},S_{2}\right)\leq a+\frac{1}{2}\log_{2}\left(\tilde{c}\;x^{2}+{\tilde{\sigma }}_{1}^{2}\right) \end{array}$$

where $\tilde{c}$ and ${\tilde{\sigma }}_{1}^{2}$ are as defined in (52), (53), and $a=\frac{1}{2}\log_{2}\left({\left(2\pi e\right)}^{2}\sigma_{S_{1}}^{2}\sigma_{S_{2}}^{2}\right)$. Due to the monotonicity of the integral and symmetry properties, we have

(59) $$\begin{array}{cc} E_{X}\left[h(S_{1}X+N_{1}|S_{2})\right] & =\;E_{X}\left[h(S_{1}X+N_{1},S_{2})\right]-h\left(S_{2}\right)\\ \; & \leq \;2\int_{0}^{\infty }\;\left(a+\frac{1}{2}\log_{2}\left(\tilde{c}\;x^{2}+{\tilde{\sigma }}_{1}^{2}\right)\right)f_{X}\left(x\right)\;dx\;-\;h\left(S_{2}\right). \end{array}$$

To evaluate the integral, we use the substitution $u=x^{2}$, the correspondence ([28], 2.6.23.4) (please note that in ([28], 2.6.23.4), the sign before F22(·;·;·) is incorrect), and the identities erfi(y)=−ıerf(ıy) and π2zerf(z)=F1112;32;−z2 ([19], 13.6.7). Applying the expression in (45) yields the bound for $E_{X}\left[h(S_{1}X+N_{1}|S_{2}\right)]$ given in Proposition 3. □

The representation in Proposition 2 as a one-dimensional integral is particularly convenient for numerical evaluations and is used in Section 5.

### 4.3. Evaluation of Equation *(8c)*

**Proposition** **4.** *Under the assumptions above, we have*

(60) $$\begin{array}{c} E_{S_{1}}[h\left(S_{1}X+N_{1}\right)]-h\left(N_{1}\right)=-\frac{1}{2\ln(2)}\exp\left(\frac{1}{P}\right)\mathrm{Ei}\left(\frac{1}{P}\right). \end{array}$$

**Proof** **of Proposition 4.** Fix $S_{1}=s_{1}$ for some $s_{1}\geq 0$. Then, $s_{1}X+N_{1}$ is a Gaussian random variable with zero mean and variance $s_{1}^{2}P+\sigma_{N_{1}}^{2}$ since *X* and $N_{1}$ are independent Gaussian random variables with positive variances *P* and $\sigma_{N_{1}}^{2}$. For the differential entropy $h(s_{1}X+N_{1})$, we obtain

(61) $$\begin{array}{c} h(s_{1}X+N_{1})=\frac{1}{2}\log_{2}\left(2\pi e\sigma_{N_{1}}^{2}P\right)+\frac{1}{2}\log_{2}\left(\frac{s_{1}^{2}}{\sigma_{N_{1}}^{2}}+\frac{1}{P}\right). \end{array}$$

Thus, we can write

(62) $$\begin{array}{c} E_{S_{1}}\left[h(S_{1}X+N_{1})\right]=h\left(N_{1}\right)+\frac{1}{2}\log_{2}\left(P\right)+\frac{1}{2}\;E_{T_{1}}\left[\log_{2}\left(T_{1}+1/P\right)\right] \end{array}$$

where $T_{1}=S_{1}^{2}/\sigma_{N_{1}}^{2}$. With the marginal pdf of $S_{1}$ and basic density transformation, we obtain the pdf $f_{T_{1}}\left(t_{1}\right)=\exp\left(-t_{1}\right)$ for $t_{1}\geq 0$ of the random variable $T_{1}$ such that we have

(63) $$\begin{array}{cc} E_{T_{1}}\left[\log_{2}\left(T_{1}+1/P\right)\right] & =\;\int_{0}^{\infty }\log_{2}\left(t_{1}+1/P\right)\exp\left(-t_{1}\right)\;dt_{1}\\ \; & =\;-\log_{2}\left(P\right)-\frac{1}{\ln(2)}\exp\left(\frac{1}{P}\right)\mathrm{Ei}\left(-\frac{1}{P}\right). \end{array}$$

The integral is solved using the substitution $u=(t_{1}+1/P)$ and integration by parts. Collecting the terms yields (60). □

## 5. Numerical Results and Discussions

We next evaluate the results of Section 4 numerically for interesting parameter regimes. To simplify notation, we denote the sum of (8a) and (8b) by $R_{\alpha }$ and the sum of (8a) and the upper bound (51) of (8b) by $R_{\alpha ,\mathrm{ub}}$, respectively. Furthermore, we denote (8c) by $R_{\beta }$. With this notation, we have for the achievable rate in Section 4

(64) $$\begin{array}{c} R\leq \;\min\left\{R_{\alpha },R_{\beta }\right\}\leq \;\min\left\{R_{\alpha ,\mathrm{ub}},R_{\beta }\right\}. \end{array}$$

Based on the representation in (16)–(18) as a one-dimensional integral, we numerically evaluate (8a). Similarly, the upper bound in (51) is numerically evaluated, and the same applies to (8c) using (60). However, the numerical evaluation of (8b) is more involved. First, we numerically calculate the convolution integral in (48) on a sufficiently-dense grid for the variables $y_{1}$ and $s_{2}$. Then, we numerically calculate the differential entropy $h(xS_{1}+N_{1},S_{2})$ using (49) based on an interpolated version of the density $f_{Y_{1}\left(x\right),S_{2}}\left(y_{1},s_{2}\right)$. Repeating these calculations for a sufficiently dense set of values *x*, we numerically calculate $E_{X}\left[h(S_{1}X+N_{1},S_{2})\right]$ using (50) and an interpolated version of the function $x\;\mapsto \;h(xS_{1}\;+\;N_{1},S_{2}).$ Combining with (45), we finally obtain (8b).

We consider a stochastically degraded secure ISAC channel, i. e., we assume that the chosen parameter values satisfy the inequality (15). Moreover, we assume that $\sigma_{N_{1}}^{2}\;>\;\sigma_{N_{2}}^{2}$, which is the interesting regime where the corresponding wiretap channel without fading does not allow secure communication. The parameter sets satisfying these conditions for which we subsequently discuss the numerical results below are given in Table 1.

**Figure 2:**
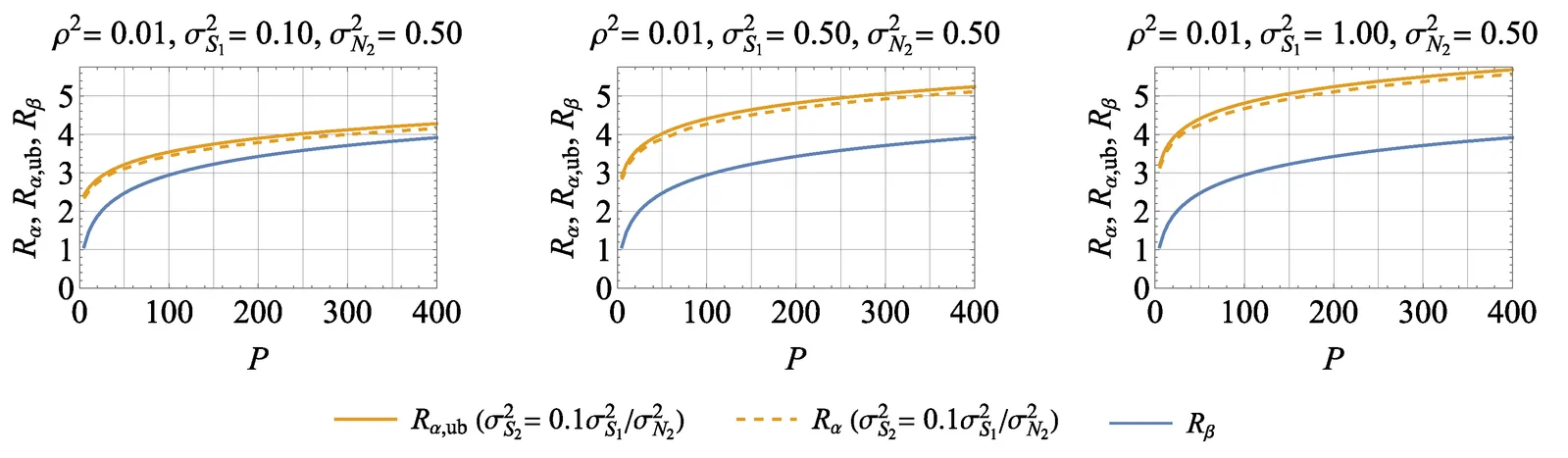
$R_{\alpha },\;R_{\alpha ,\mathrm{ub}},\;\mathrm{and}\;R_{\beta }$ for power correlation coefficient $\rho^{2}=0.01$.

**Figure 3:**
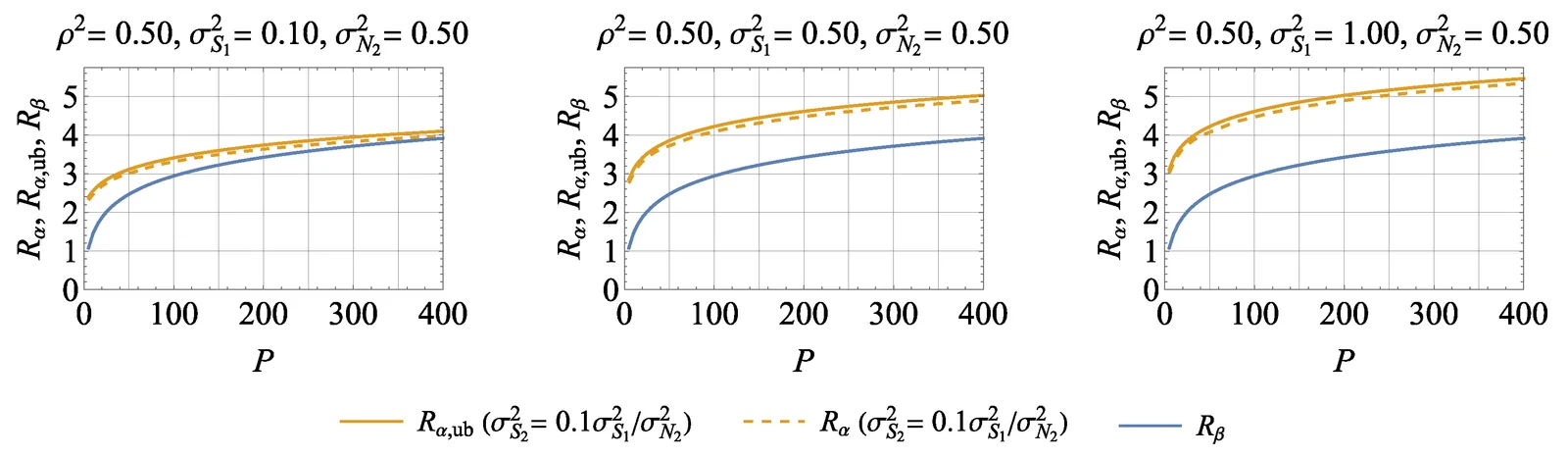
$R_{\alpha },\;R_{\alpha ,\mathrm{ub}},\;\mathrm{and}\;R_{\beta }$ for power correlation coefficient $\rho^{2}=0.50$.

**Figure 4:**
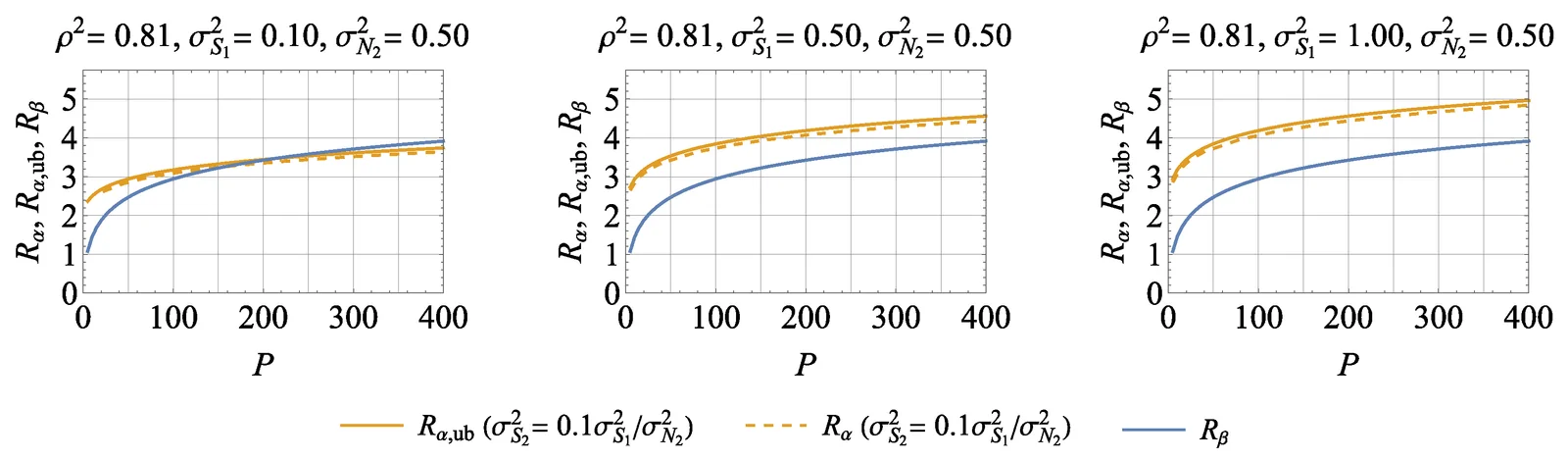
$R_{\alpha },\;R_{\alpha ,\mathrm{ub}},\;\mathrm{and}\;R_{\beta }$ for power correlation coefficient $\rho^{2}=0.81$.

**Figure 5:**
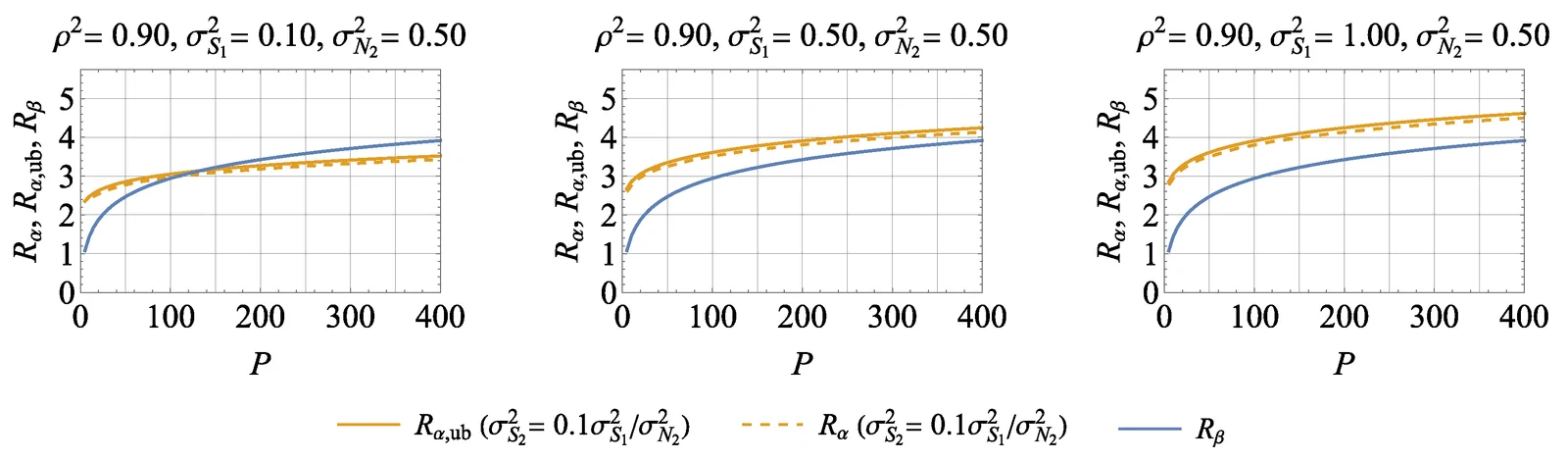
$R_{\alpha },\;R_{\alpha ,\mathrm{ub}},\;\mathrm{and}\;R_{\beta }$ for power correlation coefficient $\rho^{2}=0.90$.

We compute the results for $R_{\alpha }$, $R_{\alpha ,\mathrm{ub}}$, and $R_{\beta }$ as a function of the transmit power *P* for different values of the power correlation coefficient $\rho^{2}$. The corresponding curves are shown in Figure 2, Figure 3, Figure 4 and Figure 5. For the row of subfigures in each figure, the parameter $\sigma_{S_{1}}^{2}$ is modified from left to right, whereas $\sigma_{N_{1}}^{2}=1$, $\sigma_{N_{2}}^{2}=0.5$, and $\sigma_{S_{2}}^{2}=\sigma_{S_{1}}^{2}/10\sigma_{N_{2}}^{2}$ are fixed.

We next list our conclusions for a degraded secure ISAC channel with correlated Rayleigh fading for the parameter ranges given above, drawn from the computations mentioned above. From (60), we observe that $R_{\beta }$ is only a function of the transmit power *P* such that the curves of $R_{\beta }$ are the same in all diagrams. From (16)–(18), we observe that (8a) as a summand of $R_{\alpha }$ and $R_{\alpha ,\mathrm{ub}}$ is a function of $\rho^{2}$, *P*, and the parameter ratios $\sigma_{N_{1}}^{2}/\sigma_{S_{1}}^{2}$ and $\sigma_{N_{2}}^{2}/\sigma_{S_{2}}^{2}$. Similarly, the upper bound (51) as a summand of $R_{\alpha ,\mathrm{ub}}$ is a function of $\rho^{2}$, *P*, $\sigma_{S_{1}}^{2}$, and $\sigma_{N_{1}}^{2}$ and it does not depend on $\sigma_{S_{2}}^{2}$ and $\sigma_{N_{2}}^{2}$.

The results show that $R_{\alpha ,\mathrm{ub}}$ and $R_{\alpha }$ curves behave highly similar with a small constant gap. Thus, for most of the parameter constellations, the much-easier-to-calculate $R_{\alpha ,\mathrm{ub}}$, instead of $R_{\alpha }$, can be used to interpret the results.

Furthermore, we observe the following monotonicities: $R_{\alpha }$ increases for increasing parameters $\sigma_{S_{1}}^{2}$ and for decreasing parameters $\rho^{2}$. Moreover, we observe that increasing the power correlation $\rho^{2}$ from 0 to 0.50 has only a minor effect, whereas the impact of increasing $\rho^{2}$ from 0.50 to 0.81 is much stronger. This trend continues when $\rho^{2}$ further increases from 0.81 to 0.90.

The interesting regime in which the channel capacity is approached is when $R_{\beta }$ determines the right-hand side of (8). Figure 2, Figure 3, Figure 4 and Figure 5 show that the range of power *P* for which the channel capacity is approached stretches over all considered power values except when the power correlation coefficient $\rho^{2}$ is high combined with a low fading parameter $\sigma_{S_{1}}^{2}$, as can be observed in the leftmost diagrams of Figure 4 and Figure 5. Thus, we observe that in the low power regime, channel capacity is always approached irrespective of the values of the remaining parameters.

## 6. Conclusions

We considered a new secure ISAC model for a state-dependent wiretap channel under correlated Rayleigh fading with channel output feedback. We derived and evaluated an achievable secrecy-distortion region and demonstrated conditions where the secrecy capacity can be surpassed, unlike classical physical layer security methods, which provides fundamental insights essential for designing optimal secure ISAC systems for future communication systems. We remark that extensions of our model to consider active attacks, as in, e.g., [29], and evaluations for more practical ISAC channel models, as in, e.g., [30], are also important for secure ISAC research.

## Figures and Tables

**Table 1:** Parameter sets for numerical calculations.

$\rho^{2}\in \left\{0.01,0.50,0.81,0.90\right\}$	
$\sigma_{N_{1}}^{2}=1$	$\sigma_{N_{2}}^{2}=0.50$
$\sigma_{S_{1}}^{2}\in \left\{0.10,0.50,1.00\right\}$	$\sigma_{S_{2}}^{2}=\sigma_{S_{1}}^{2}/10\sigma_{N_{2}}^{2}$

## Data Availability

The original contributions presented in this study are included in the article. Further inquiries can be directed to the corresponding author.
