# Correction: Mittelbach et al. Sensing-Assisted Secure Communications over Correlated Rayleigh Fading Channels. *Entropy* 2025, *27*, 225

**DOI:** 10.3390/e28040378

**Published:** 2026-03-27

**Authors:** Martin Mittelbach, Rafael F. Schaefer, Matthieu Bloch, Aylin Yener, Onur Günlü

**Affiliations:** 1 Chair of Information Theory and Machine Learning, Technische Universität Dresden, 01062 Dresden, Germany; martin.mittelbach@tu-dresden.de (M.M.); rafael.schaefer@tu-dresden.de (R.F.S.); 2 School of Electrical and Computer Engineering, Georgia Institute of Technology, Atlanta, GA 30332, USA; matthieu.bloch@ece.gatech.edu; 3 Department of Electrical and Computer Engineering, The Ohio State University, Columbus, OH 43210, USA; yener@ece.osu.edu; 4 Information Theory and Security Laboratory (ITSL), Linköping University, 58183 Linköping, Sweden

In Proposition 1 of [1], the sufficient condition for stochastic degradedness was stated incorrectly. The correct condition corresponding to $S_{1}^{2}/\sigma_{N_{1}}^{2}$ being stochastically larger than $S_{2}^{2}/\sigma_{N_{2}}^{2}$ is ${\bar{F}}_{S_{1}^{2}}\;\left(s\;\sigma_{N_{1}}^{2}\right)\geq {\bar{F}}_{S_{2}^{2}}\;\left(s\;\sigma_{N_{2}}^{2}\right)$ for all s≥0. Therefore, Equation (9) should be replaced with

(9) $${\bar{F}}_{S_{1}^{2}}\;\left(s\;\sigma_{N_{1}}^{2}\right)\geq {\bar{F}}_{S_{2}^{2}}\;\left(s\;\sigma_{N_{2}}^{2}\right).$$

For the bivariate Rayleigh fading model in Section 3.2, using the complementary cdf ${\bar{F}}_{S_{j}^{2}}\left(u\right)=\exp\left(-u/\sigma_{S_{j}}^{2}\right)$, u≥0, j∈{1,2}, the corrected condition yields $\sigma_{S_{2}}^{2}/\sigma_{N_{2}}^{2}\leq \sigma_{S_{1}}^{2}/\sigma_{N_{1}}^{2}$. Therefore, Equation (15) should be replaced with

(15) $$\frac{\sigma_{S_{2}}^{2}}{\sigma_{N_{2}}^{2}}\leq \frac{\sigma_{S_{1}}^{2}}{\sigma_{N_{1}}^{2}}.$$

As a consequence, among the parameter sets originally listed in Table 1, the following parameter sets must be removed: $\left(\sigma_{N_{2}}^{2}=0.10,\;\sigma_{S_{2}}^{2}=\sigma_{S_{1}}^{2}/\sigma_{N_{2}}^{2}\right)$, $\left(\sigma_{N_{2}}^{2}=0.10,\;\sigma_{S_{2}}^{2}=\sigma_{S_{1}}^{2}/10\sigma_{N_{2}}^{2}\right)$ and $\left(\sigma_{N_{2}}^{2}=0.50,\;\sigma_{S_{2}}^{2}=\sigma_{S_{1}}^{2}/\sigma_{N_{2}}^{2}\right)$, for all values of $\rho^{2}\in \left\{0.01,0.50,0.81,0.90\right\}$ and $\sigma_{S_{1}}^{2}\in \left\{0.10,0.50,1.00\right\}$.

Accordingly, based on these changes, the following changes have been made to the original publication:

1. In Table 1, only the degraded-case parameter sets should remain, namely,

**Table 1:** Parameter sets for numerical calculations.

$\rho^{2}\in \left\{0.01,0.50,0.81,0.90\right\}$	
$\sigma_{N_{1}}^{2}=1$	$\sigma_{N_{2}}^{2}=0.50$
$\sigma_{S_{1}}^{2}\in \left\{0.10,0.50,1.00\right\}$	$\sigma_{S_{2}}^{2}=\sigma_{S_{1}}^{2}/10\sigma_{N_{2}}^{2}$
2. Figures 2–5 must be updated by removing all subfigures in the top row and, in each remaining bottom-row subfigure, removing the curves corresponding to $\sigma_{S_{2}}^{2}=\sigma_{S_{1}}^{2}/\sigma_{N_{2}}^{2}$.
3. In the legends of Figures 2–5, the entries corresponding to $\sigma_{S_{2}}^{2}=\sigma_{S_{1}}^{2}/\sigma_{N_{2}}^{2}$ must be removed.
4. In Section 5, in the paragraph beginning with “We consider a stochastically degraded secure ISAC channel …, ” the formulation regarding the choice of the considered parameter sets for the degraded-case should be updated and stated more precisely.
5. In Section 5, the paragraph beginning with “We compute the results for $R_{\alpha }$, $R_{\alpha ,\mathrm{ub}}$, and $R_{\beta }$ …,” should be updated so that it no longer states that $\sigma_{N_{2}}^{2}$ is modified from top to bottom and that $\sigma_{S_{2}}^{2}$ is modified within a subfigure.
6. In Section 5, the discussion should be updated by removing claims that rely on the deleted parameter variations in $\sigma_{N_{2}}^{2}$ and in the two values of $\sigma_{S_{2}}^{2}$. In particular, in the paragraph beginning with “We next list our conclusions for a degraded secure ISAC channel …,” the following sentence should be removed:
  - “The numerical results also indicate that (8b) as a summand of $R_{\alpha }$ does not depend on $\sigma_{S_{2}}^{2}$ and $\sigma_{N_{2}}^{2}$.”.
  In the paragraph beginning with “Furthermore, we observe the following monotonicities …,” the sentence
  - “Furthermore, we observe the following monotonicities: $R_{\alpha }$ increases for increasing parameters $\sigma_{S_{1}}^{2}$ or $\sigma_{N_{2}}^{2}$ and for decreasing parameters $\sigma_{S_{2}}^{2}$ or $\rho^{2}$.”
  should be replaced with the sentence
  - “Furthermore, we observe the following monotonicities: $R_{\alpha }$ increases for increasing parameter $\sigma_{S_{1}}^{2}$ and for decreasing parameter $\rho^{2}$.”.
  Moreover, in the last paragraph beginning with “The interesting regime where the channel capacity is approached …,” the sentences
  - “The range for the power *P* where $R_{\beta }$ determines (8) increases with increasing $\sigma_{S_{1}}^{2}$ or $\sigma_{N_{2}}^{2}$ and decreasing $\sigma_{S_{2}}^{2}$ or $\rho^{2}$. For low correlation $\rho^{2}$, this range stretches over all considered power values for almost all parameter constellations, whereas for highly correlated fading coefficients it shrinks to low power values. Thus, in the low power regime, channel capacity is approached irrespective of the values of the remaining parameters.”
  should be replaced with the sentences
  - “Figures 2–5 show that the range of power *P* for which the channel capacity is approached stretches over all considered power values except when the power correlation coefficient $\rho^{2}$ is high combined with a low fading parameter $\sigma_{S_{1}}^{2}$, as can be observed in the leftmost diagrams of Figures 4 and 5. Thus, we observe that in the low power regime, channel capacity is always approached irrespective of the values of the remaining parameters.”.

The authors state that the scientific conclusions are unaffected. This correction was approved by the Academic Editor. The original publication has also been updated.
